# Supplementary material for: Cognitive Deficits in the Acute Phase of COVID-19: A Review and Meta-Analysis
Source: J Clin Med. 2023 Jan 18;12(3):762. doi: 10.3390/jcm12030762 (PMC9917639; doi:10.3390/jcm12030762)
Supplement: Supplementary file 1 [file jcm-12-00762-s001.zip › jcm-2080323-supplementary.pdf]

Table S1. Studies included in the logistic regression.

Articles	Severity	Sample Size	Age (Mean or Median)	Time Since Disease Onset (Mean or Median Days)	MoCA (Mean)	MoCA (SD)	MMSE	MMSE (SD)	TICS-M (Mean)	Cognitive Impairment (0 = no, 1 = yes)
Alemanno et al., 2021	Severe	31	60 ± 8.9	Inpatients *	21.65	5.23				1
Alemanno et al., 2021	Intermed.	56	69 ± 5.7	Inpatients *	17.28	6.97				1
Beaud et al., 2020	Severe	13	65 ± 7.6	Inpatients *	19.7	7.5				1
Blazhenets et al.,2021	Intermed.	8	66 ± 14.2	28 ± 14.6	19.13	4.5				1
Di Pietro et al.,2021	Severe	8	60 ± 12.1	57 ± 20.7	27.15					0
Ermis et al., 2021	Intermed.	13	61 ± 13.3	Inpatients *	23	5.12				1
Heyns et al., 2021	Intermed.	38	na	Inpatients *	21					1
Hosp et al., 2021	Intermed.	26	65 ± 14.4	18.4 ± 2.3	21.8					1
Jain et al., 2021	Intermed.	16	na	Inpatients *	23					1
Martillo et al., 2021	Severe	30	54 ± 12.9	30	20 &					0
Monti et al., 2021	Severe	37	56 ± 10.5	61 (IQR 51–71)			22 &			0
Negrini et al., 2020	Intermed.	4	60 ± 26.2	At least 30			28.5			0
Negrini et al., 2020	Severe	5	61 ± 4.4	At least 30			24.83			0
Ortelli et al., 2021	Intermed.	12	67 ± 9.6	81 ± 9.2	17.8	5.3				1
Patel et al., 2021	Severe	77	62 ± 15.7	Inpatients *	20,29	5,47				1
Pirker-Kees et al.,2021	Intermed.	7	79 ± 8.4	15 ± 6.2	20	5				1
Pistarini et al., 2021	Intermed.	40	64 ± 11.9	Inpatients *	21,97	5,42				1
Rousseau et al., 2021	Severe	32	62 (IQR 49–68)	94 (IQR 90–101)	27					0
Solaro et al., 2021	Intermed.	32	54 ± 4.8	Inpatients *	23,5	4.24				1
Udina et al., 2021	Intermed.	13	78 ± 8.1	Inpatients *	21,6	5,3				1
Udina et al., 2021	Severe	20	58 ± 7.9	Inpatients *	22,9	4,7				1

& Telephone version; * Mean time from disease onset not available but assessment was performed in inpatient in the acute phase of the infection. MoCA: Montreal scale of Cognitive Assessment; MMSE: Mini Mental State Examination; TICS: Telephone Interview for Cognitive Status; TICS-M: Telephone Interview for Cognitive Status Modified; Mild: patients not hospitalized; Intermed.: patients in intermediate or intensive care.

Table S2. Mean age difference between studies with hospitalized patients with (severe) and without (intermed) mechanical ventilation.

Independent Samples *t*-Test			
	**t**	**df**	***p***
Age	2.352	19	0.030

Note: Student's *t*-test.

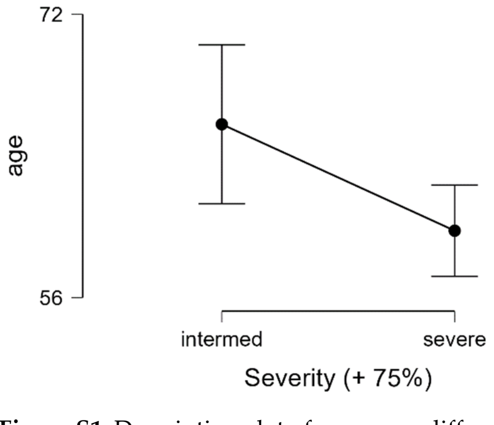

Figure S1. Descriptive plot of mean age difference between studies with hospitalized patients with (severe) and without (intermed) mechanical ventilation.
